# SERINC5 Potently Restricts Retrovirus Infection *In Vivo*

**DOI:** 10.1128/mBio.00588-20

**Published:** 2020-07-14

**Authors:** Uddhav Timilsina, Supawadee Umthong, Brian Lynch, Aimee Stablewski, Spyridon Stavrou

**Affiliations:** a Department of Microbiology and Immunology, Jacobs School of Medicine and Biomedical Sciences, University at Buffalo, Buffalo, New York, USA; b Department of Molecular and Cell Biology, Roswell Park Comprehensive Cancer Center, Buffalo, New York, USA; University of Trento; Albert Einstein College of Medicine

**Keywords:** SERINC3, SERINC5, antiretroviral response, glycosylated gag, *in vivo* model

## Abstract

The serine incorporator (SERINC) proteins are multipass transmembrane proteins that affect sphingolipid and phosphatidylserine synthesis. Human SERINC5 and SERINC3 were recently shown to possess antiretroviral activity for a number of retroviruses, including human immunodeficiency virus (HIV), murine leukemia virus (MLV), and equine infectious anemia virus (EIAV). In the case of MLV, the glycosylated Gag (glyco-Gag) protein was shown to counteract SERINC5-mediated restriction in *in vitro* experiments and the viral envelope was found to determine virion sensitivity or resistance to SERINC5. However, nothing is known about the *in vivo* function of SERINC5. Antiretroviral function of a host factor *in vitro* is not always associated with antiretroviral function *in vivo*. Using SERINC5^−/−^ mice that we had generated, we showed that mouse SERINC5 (mSERINC5) restriction of MLV infection *in vivo* is influenced not only by glyco-Gag but also by the retroviral envelope. Finally, we also examined the *in vivo* function of the other SERINC gene with known antiretroviral functions, SERINC3. By using SERINC3^−/−^ mice, we found that the murine homologue, mSERINC3, had no antiretroviral role either *in vivo* or *in vitro*. To our knowledge, this report provides the first data showing that SERINC5 restricts retrovirus infection *in vivo* and that restriction of retrovirus infectivity *in vivo* is dependent on the presence of both glyco-Gag and the viral envelope.

## Abstract (precis)

This study examined for the first time the *in vivo* function of the serine incorporator (SERINC) proteins during retrovirus infection. SERINC3 and SERINC5 (SERINC3/5) restrict a number of retroviruses, including human immunodeficiency virus 1 (HIV-1) and murine leukemia virus (MLV), by blocking their entry into cells. Nevertheless, HIV-1 and MLV encode factors, Nef and glycosylated Gag, respectively, that counteract SERINC3/5 *in vitro*. We recently developed SERINC3 and SERINC5 knockout mice to examine the *in vivo* function of these genes. We found that SERINC5 restriction is dependent on the absence of glycosylated Gag and the expression of a specific viral envelope glycoprotein. On the other hand, SERINC3 had no antiviral function. Our findings have implications for the development of therapeutics that target SERINC5 during retrovirus infection.

## INTRODUCTION

Cells have developed various restriction factors that counteract infection by inhibiting different points of the viral life cycle. Among these host restriction factors are the serine incorporator (SERINC) proteins. The SERINC family of proteins consists of 5 members (SERINC1 to SERINC5) and is conserved in all eukaryotes. They are all transmembrane proteins and are implicated in sphingolipid and phosphatidylserine biogenesis (1). Human SERINC3 (hSERINC3) and SERINC5 can inhibit a variety of retroviruses *in vitro*, including human immunodeficiency virus 1 (HIV-1) and murine leukemia virus (MLV) (2–5), with SERINC5 being more potently antiretroviral than SERINC3 (3, 4, 6). SERINC3 and SERINC5 (SERINC3/5) are incorporated into budding virions (3, 4) and block the step of HIV envelope fusion and pore formation with the target cell membrane (7, 8).

MLV is divided into different subgroups on the basis of host range (9). Ecotropic MLVs, such as Friend MLV (F-MLV), infect only mouse cells; amphotropic MLVs, such as 4070A, infect both human and mouse cells; and xenotropic MLVs infect nonmurine cells (10). Glycosylated Gag (glyco-Gag) is a viral protein expressed by both the ecotropic and amphotropic MLVs (11). Glyco-Gag expression is initiated from a CUG upstream of the polyprotein’s AUG, is highly glycosylated (12–15), and is cleaved into two fragments by an unknown protease (14, 16). The N-terminal fragment is a transmembrane protein, and the C-terminal fragment is secreted from the infected cell (12, 17, 18). While glyco-Gag is considered to be dispensable *in vitro*, glyco-Gag-deficient viruses exhibit lower infectivity *in vivo* (19–21). Glyco-Gag is important *in vivo*, because it protects the virus from both the deleterious effects of mouse apolipoprotein B editing complex 3 (APOBEC3 [A3]), a potent antiretroviral factor, and induction of the type I interferon (IFN) response (11, 22–24). Glyco-Gag antagonizes the antiviral function of SERINC3 and SERINC5 *in vitro* by blocking the incorporation of SERINC proteins into the budding virions, leading to their lysosomal degradation (3, 4, 25, 26). Whether SERINC5 restricts retrovirus infection *in vivo* in a glyco-Gag-dependent manner is currently unknown.

While much work has been performed to understand the role of SERINC proteins in retrovirus infection *in vitro*, nothing is known about the antiretroviral function of these proteins *in vivo*. A host factor that has an antiretroviral function *in vitro* does not necessarily mean that it can restrict retrovirus infection *in vivo*. For example, SAMHD1, a potent retroviral factor, did not affect retrovirus infection *in vivo* (27). Here, for the first time, we examine the antiretroviral effect of SERINC5 *in vivo* and show that mouse SERINC5 (mSERINC5) restricts MLV infection *in vivo*. Moreover, SERINC5 restriction *in vivo* is influenced not only by the presence of glyco-Gag but also by the virus envelope. SERINC5 had no effect on F-MLV infectivity even when glyco-Gag was mutated; however, it was only when we replaced the F-MLV envelope with the amphotropic MLV 4070A envelope that we found that SERINC5 restricted MLV infection in a glyco-Gag-dependent manner. Finally, unlike human SERINC3, mouse SERINC3 has no antiretroviral function either *in vivo* or *in vitro*.

## RESULTS

### Murine SERINC3 and SERINC5 are expressed in murine leukocytes and are not induced by F-MLV infection.

**FIG 1:**
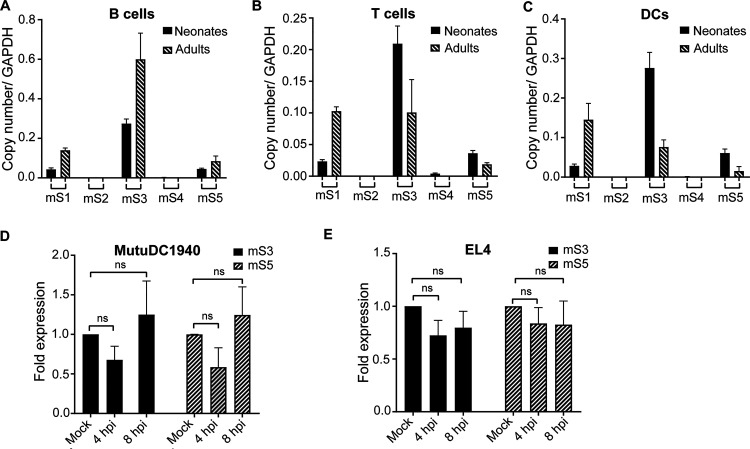
mSERINC3 and SERINC5 are constitutively expressed in murine leukocytes and are not induced by F-MLV infection. (A to C) mSERINC1-5 RNA copy number relative to GAPDH from (A) B cells (CD45R/B220^+^), (B) T cells (CD3^+^), and (C) dendritic cells (DCs) (CD11c^+^) sorted from peripheral blood mononuclear cells isolated from the blood of C57BL/6 neonates or adults (*n* = 3 per age group). (D and E) Fold expression changes in mSERINC3 and mSERINC5 transcripts relative to mock-infected cells, normalized to GAPDH, in (D) MutuDC1940 and (E) EL4 cells infected with F-MLV for 4 h or 8 h. “Mock” indicates mock infection (PBS). Data shown represent averages of results from 3 independent experiments. Statistical significance was determined by one-way analysis of variance (ANOVA) and Tukey’s test. All results are presented as means ± standard deviations (SD). ns, not significant; mS1 to mS5, mouse SERINC1 to SERINC5, respectively; hpi, hour postinfection.

To elucidate whether the mouse SERINC genes are expressed in leukocytes, cells that are naturally infected by F-MLV in mice (23, 28, 29), we isolated peripheral blood mononuclear cells (PBMCs) from 3 adult and 3 neonate BL/6 mice (C57BL/6N-wild-type mice) and performed cell sorting for T cells, B cells, and dendritic cells (DCs). Transcription levels of SERINC genes were determined by real-time quantitative PCR (RT-qPCR). We observed that only mSERINC1 RNA, mSERINC3 RNA, and mSERINC5 RNA were detected in both adults and neonates, while mSERINC2 and mSERINC4 were absent from these cell populations (Fig. 1A to C). To determine the effect of F-MLV infection on mSERINC3 and mSERINC5 transcription, we infected EL-4 cells (T lymphoblast cell line) and MutuDC1940 cells (a murine DC cell line [30]) with F-MLV wild type (F-MLV WT) (0.1 multiplicity of infection [MOI]) followed by RT-qPCR to determine mSERINC3 and mSERINC5 transcription levels over time. No changes in the transcription levels of mSERINC5 and mSERINC3 were observed during infection (Fig. 1D and E). Thus, we concluded that F-MLV infection does not affect mSERINC3 and mSERINC5 transcription. To determine if type I interferons (IFNs) induce the expression of SERINC genes *in vivo*, we treated BL/6 mice with polyinosinic:polycytidiylic acid [poly(I·C)] to mimic type I IFN stimulation. No increases were detected in the expression levels of any of the SERINC genes in the spleens of the poly(I·C)-treated mice (see Fig. S1 in the supplemental material). Thus, mouse SERINC genes, similarly to human SERINC genes (3), are not upregulated by type I IFNs.

10.1128/mBio.00588-20.1 FIG S1 mSERINC genes are not stimulated by poly(I·C) treatment. Data represent fold changes in expression of mSERINC1, mSERINC2, mSERINC3, mSERINC4, mSERINC5, and ISG15 genes in the spleens of C57BL/6 mice treated with 8 mg/kg of poly(I·C) normalized to GAPDH and compared to the expression levels observed in the mock-treated (PBS) mice. All results are presented as means ± SD. Statistical significance was determined by unpaired (two-tailed) *t* test. **, *P* < 0.01 (*n* = 4 in each group). Download FIG S1, TIF file, 0.5 MB. Copyright © 2020 Timilsina et al. 2020 Timilsina et al. This content is distributed under the terms of the Creative Commons Attribution 4.0 International license.

### Generation of the SERINC5^−/−^ mice.

**FIG 2:**
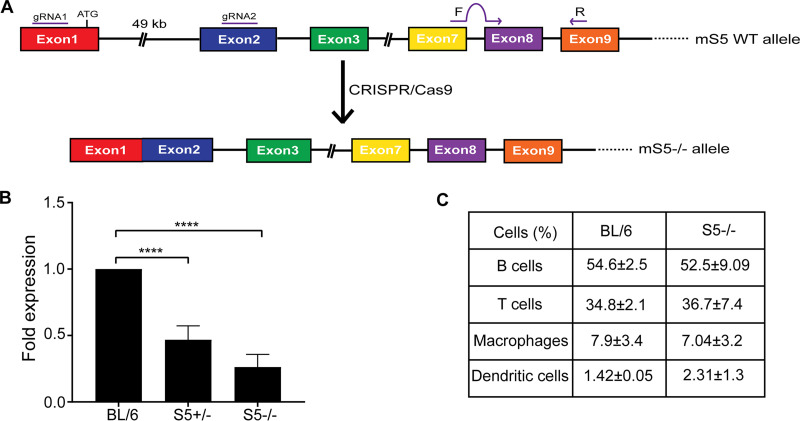
SERINC5^−/−^ mice have lower levels of mSERINC5 and retain normal populations of leukocytes. (A) Schematic of the SERINC5 allele in the SERINC5^−/−^ mice. Two guide RNAs (gRNAs) were used to target exons 1 and 2. The genomic fragment between the two gRNAs was excised using CRISPR/Cas9 technology, resulting in the abrogation of the SERINC5 gene. Locations of the SERINC5 primers used for RT-PCR analysis are indicated by the “F” and “R” purple arrows. (B) Fold change of SERINC5 expression in the SERINC5^−/−^ and SERINC5^+/−^ mice relative to C57BL/6 mice. SERINC5 RNA levels in the SERINC5^−/−^ mice (*n* = 5), SERINC5^+/−^ mice (*n* = 6), and C57BL/6 mice (*n* = 6) were determined by RT-qPCR and normalized to β-actin. (C) PBMCs from 4 C57BL/6 mice and 6 SERINC5^−/−^ mice were stained with anti-CD45R/B220 (B cells), anti-CD3 (T cells), anti-F4/80 (Macrophages), and anti-CD11c (Dendritic cells) antibodies and subjected to FACS analysis. Values are presented as means ± SD. There were no significant differences in the percentages of T cells, B cells, macrophages, and dendritic cells among the C57BL/6 and SERINC5^−/−^ mice. For the data shown in panel B, statistical significance was determined by one-way ANOVA and Tukey’s test; for the data shown in panel C, statistical significance was determined by an unpaired *t* test, **, *P* < 0.01; ****, *P* < 0.0001. S5, SERINC5; BL/6, C57BL/6.

As SERINC5 is the most potent antiretroviral member of the SERINC family of proteins (3–5), we generated SERINC5-deficient mice in the C57BL/6 (BL/6) background by targeting SERINC5 for deletion using clustered regularly interspersed short palindromic repeat (CRISPR)/CRISPR-associated 9 (Cas9) technology (Fig. 2A). Following electroporation performed to generate CRISPR knockout mice, we identified two founder lines, both of which were successful breeders. One founder line was discovered to have a sizable deletion (∼49 kb) in the mouse SERINC5 gene comprising parts of exon 1 (including the initiation codon), exon 2, and the entire intron between these two exons (Fig. S2). We isolated RNA from the tails of SERINC5^+/+^ (BL/6) mice, SERINC5^+/−^ (heterozygous) mice, and SERINC5^−/−^ (homozygous knockout) mice and found that the SERINC5^−/−^ mice had significantly lower levels of SERINC5 transcripts than the SERINC5^+/+^ mice, while the heterozygotes (+/−) had SERINC5 transcript levels intermediate between those seen with the SERINC5^+/+^ and the SERINC5^−/−^ mice (Fig. 2B). SERINC5^−/−^ mice had no apparent physical, developmental, reproductive, or behavioral defects, and fluorescence-activated cell sorter (FACS) analysis demonstrated that the SERINC5^−/−^ mice and BL/6 mice had similar levels of DCs, B cells, T cells, and macrophages (Fig. 2C).

10.1128/mBio.00588-20.2 FIG S2 Schematic of the genetic lesion created by CRISPR/Cas9 in the SERINC5^−/−^ mice. The sequence comprised of parts of exon 1 (nucleotides 112 to 150; highlighted in blue) and exon 2 (nucleotides 151 to 218; highlighted in blue) along with the entire intron (49.73 kb) between these two exons was subjected to CRISPR/Cas9 deletion in SERINC5^−/−^ mice. The position of the initiation codon is underlined. Download FIG S2, TIF file, 0.7 MB. Copyright © 2020 Timilsina et al. 2020 Timilsina et al. This content is distributed under the terms of the Creative Commons Attribution 4.0 International license.

### Murine SERINC3 and SERINC5 have no effect on ecotropic MLV infection *in vitro*.

**FIG 3:**
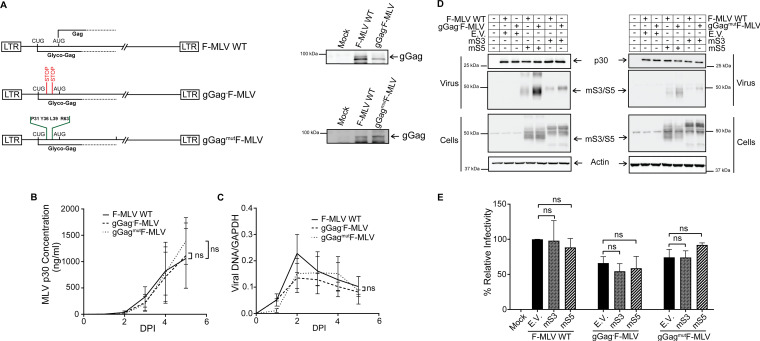
mSERINC3 and mSERINC5 have no effect on ecotropic MLV infection *in vitro*. (A) Schematic diagram of glyco-Gag (gGag) mutant MLV constructs. Positions of translation start codons (CUG for glyco-Gag and AUG for Gag) and the two stop codons inserted at amino acids 32 and 55 within the glyco-Gag reading frame to generate gGag^−^F-MLV are shown. In the case of the gGag^mut^F-MLV construct, glyco-Gag residues P31, Y36, L39, and R63 were mutated to alanine (A). Shown on the right are immunoblots of lysates from 293T cells transfected with F-MLV WT, gGag^−^F-MLV, and gGag^mut^F-MLV constructs and probed with a goat anti-MLV antibody for the detection of gGag. (B and C) Growth curves of the gGag mutant viruses. NIH 3T3 cells were infected with F-MLV WT, gGag^−^F-MLV, and gGag^mut^F-MLV (MOI 0.1). Virus replication was determined by performing p30 (CA) ELISAs to determine virus levels in the culture supernatants at the indicated time points (B) and by RT-qPCR for MLV DNA levels normalized to GAPDH in the infected NIH 3T3 (C). (D) mSERINC3 and mSERINC5 incorporation into the budding virions is glyco-Gag dependent. 293T cells were cotransfected with F-MLV WT/gGag^−^F-MLV/gGag^mut^F-MLV and mSERINC3, mSERINC5, or empty vector as indicated. At 48 h posttransfection, cells and released virus in the culture media were harvested and the indicated proteins were analyzed by immunoblotting using anti-MLV p30, anti-HA (for detection of mSERINC3 and mSERINC5) and anti-β-actin antibodies. (E) mSERINC3 and mSERINC5 do not affect F-MLV infectivity *in vitro*. Mus dunni cells were infected with equal amounts of 293T-derived F-MLV WT or gGag^−^F-MLV or gGag^mut^F-MLV virus produced in the presence of mSERINC3, mSERINC5, or empty vector. Genomic DNA was isolated 5 h postinfection (hpi), and MLV DNA levels were measured by RT-qPCR. Viral DNA levels normalized to GAPDH were used to calculate the percentage (%) of relative infectivity with respect to F-MLV WT virus produced in the presence of empty vector. All results are presented as means ± SD. Statistical significance was determined by unpaired (two-tailed) *t* test for data points at 5 dpi (B and C) and by one-way ANOVA and Tukey’s test (E). ns, not significant. Results are shown for *n* = 3 independent experiments in panels A to E; representative immunoblotting results are shown in panels A and D. mS3, mouse SERINC3; mS5, mouse SERINC5; E.V., empty vector; gGag, glyco-Gag.

While the antiretroviral role of human SERINC3 and SERINC5 has been extensively examined (3–5), there is little information regarding the role of mSERINC3 and mSERINC5. To examine the effect of glyco-Gag on mSERINC3 and mSERINC5 during infection, we generated two F-MLV infectious clones with mutations in the glyco-Gag gene (Fig. 3A), including one with two stop codons at amino acids 32 and 55 of glyco-Gag (gGag^−^F-MLV); previous work showed that introducing stop codons at these residues affected only glyco-Gag translation and not Gag translation (5). For the second virus (gGag^mut^F-MLV), residues P31, Y36, L39, and R63, which are critical for the anti-SERINC5 function of glyco-Gag, were mutated to alanine (5, 25, 31). NIH 3T3 cells, which express very low levels of mouse SERINC3 and SERINC5 (Fig. S3), were infected at MOI of 0.1 with gGag^−^F-MLV, gGag^mut^F-MLV, and F-MLV WT, and virus yields were determined at various times postinfection by p30 core antigen enzyme-linked immunosorbent assay (p30-CA ELISA), while MLV DNA levels in the infected cells were measured by RT-qPCR. gGag^−^F-MLV and gGag^mut^F-MLV did not differ from the F-MLV WT in either p30 or MLV DNA levels (Fig. 3B and C); thus, glyco-Gag is not essential for ecotropic MLV replication and infectivity *in vitro* (5, 20, 32, 33). Glyco-Gag blocks human SERINC5 and SERINC3 incorporation into nascent virions (3, 4, 34). To determine whether the glyco-Gag mutant viruses that we generated would affect the incorporation of mSERINC3 and mSERINC5 into budding MLV particles, we cotransfected 293T cells with either F-MLV WT or the F-MLV constructs with mutations in the glyco-Gag gene and with either mSERINC3 or mSERINC5. The virus released was examined for mSERINC3 and mSERINC5 content by Western blotting. We found that the gGag^−^F-MLV and gGag^mut^F-MLV particles had higher levels of mSERINC3 and mSERINC5 incorporated into their virions than the F-MLV WT particles (Fig. 3D). Thus, we concluded that glyco-Gag blocks mSERINC3 and mSERINC5 incorporation into the nascent virions.

10.1128/mBio.00588-20.3 FIG S3 NIH 3T3 cell lines express low levels of mouse SERINC3/5. (A and B) mSERINC3 (A) and mSERINC5 (B) RNA copy numbers relative to GAPDH from T cells (CD3^+^) obtained as described for Fig. 1B and the NIH 3T3 cells. Results are shown for *n* = 3 independent experiments. All results are presented as means ± SD. Statistical significance was determined by an unpaired (two-tailed) *t* test. **, *P* < 0.01. Download FIG S3, TIF file, 0.8 MB. Copyright © 2020 Timilsina et al. 2020 Timilsina et al. This content is distributed under the terms of the Creative Commons Attribution 4.0 International license.

We next examined the effect of mSERINC3 and mSERINC5 on F-MLV infection. Previous work had shown that although hSERINC3 and hSERINC5 did not affect virus release from producer cells, they blocked MLV infectivity in a glyco-Gag-dependent manner in target cells (3–5, 25, 34). To determine whether mSERINC3 and mSERINC5 packaged in the MLV particles blocked MLV infection, we infected Mus dunni cells with equal amounts of F-MLV WT, gGag^−^F-MLV, and gGag^mut^F-MLV generated in the presence of either mSERINC3 or mSERINC5. DNA was isolated from the infected cells, and viral DNA levels were measured by RT-qPCR. Interestingly, F-MLV infectivity was unaffected regardless of the levels of mSERINC3 or mSERINC5 incorporated into the nascent virions (Fig. 3E), demonstrating that while mSERINC3 and mSERINC5 were excluded from F-MLV in a glyco-Gag-dependent manner, these two host factors had no effect on F-MLV infectivity.

### Murine SERINC5 does not restrict ecotropic MLV infection *in vivo*.

**FIG 4:**
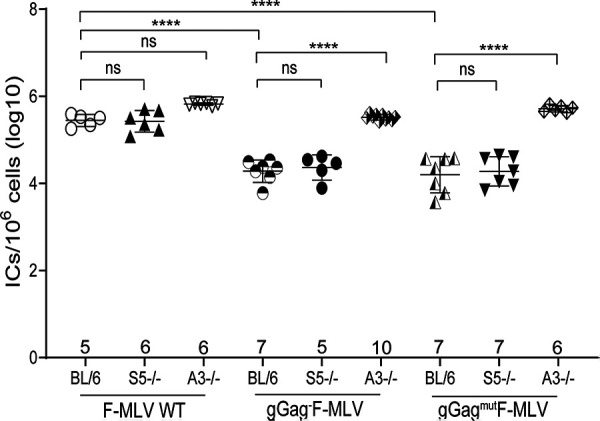
SERINC5 has no effect on ecotropic MLV infection *in vivo*. Newborn mice were infected with F-MLV WT, gGag^−^F-MLV, and gGag^mut^F-MLV virus, and virus titers in the spleens were measured 10 days postinfection. Each point represents the titer obtained from an individual mouse, and the average for each group is shown by a horizontal line. Mice were derived from 2 to 3 litters per genotype; the C57BL/6 and SERINC5^−/−^ mice were littermates. Numbers of mice used in each group are indicated on the *x* axis. All results are presented as means ± SD. Statistical significance was determined by one-way ANOVA and Tukey’s test. ****, *P* < 0.0001; ns, not significant. BL/6, C57BL/6; A3, APOBEC3; gGag, glyco-Gag; ICs, infectious centers.

While mSERINC3 and mSERINC5 did not affect F-MLV infectivity when glyco-Gag was mutated, we speculated that we might observe something different *in vivo*. *In vitro* data do not necessarily reflect what happens *in vivo.* MLV infects a variety of cells during *in vivo* infections, and involvement of the immune system, due to its activation by MLV infection, might result in changes in the levels of host genes such as the SERINC5 gene that might affect the outcome of infection. To determine the role of SERINC5 in retrovirus infection *in vivo*, we infected the SERINC5^−/−^ mice we developed with F-MLV WT, gGag^−^F-MLV, and gGag^mut^F-MLV, and virus titers in their spleens were measured at 10 days postinfection (dpi). We found that the BL/6 (SERINC5^+/+^) mice and SERINC5^−/−^ mice infected with F-MLV WT had similar virus titers in their spleens at 10 dpi (Fig. 4). Interestingly, gGag^−^F-MLV and gGag^mut^F-MLV also replicated to similar levels in the spleens of the BL/6 and SERINC5^−/−^ mice, albeit both viruses grew at significantly lower levels than F-MLV WT (Fig. 4).

As a positive control for our *in vivo* experiments, we used APOBEC3^−/−^ (A3^−/−^) mice, where a glyco-Gag mutant MLV has been shown to replicate similarly to wild-type MLV in the spleens of the infected mice (11, 22, 24). We infected APOBEC3^−/−^ mice with F-MLV WT, gGag^−^F-MLV, and gGag^mut^F-MLV, and, similarly to what had been previously reported (11, 22, 24), the three viruses replicated to similar levels in the spleens of the infected APOBEC3^−/−^ mice (Fig. 4).

Thus, we concluded that in the context of the ecotropic F-MLV, SERINC5, unlike APOBEC3, exerts no *in vivo* antiviral function regardless of the presence of glyco-Gag.

### The amphotropic MLV envelope renders MLV vulnerable to SERINC5 restriction in a glyco-Gag-dependent manner *in vitro*.

**FIG 5:**
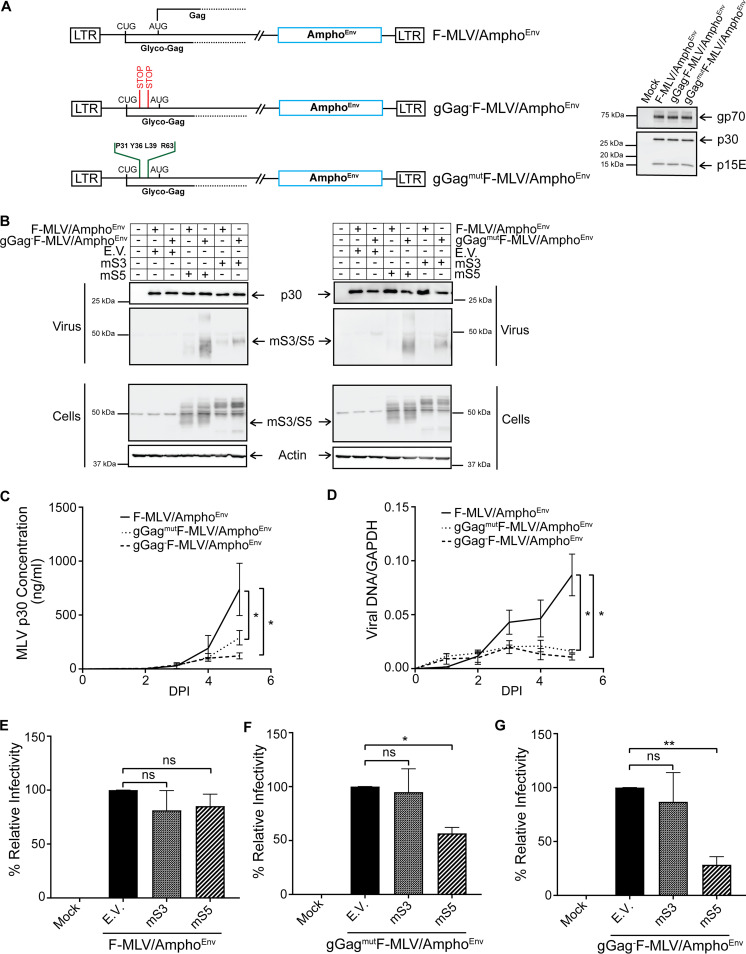
Amphotropic envelope renders MLV sensitive to mSERINC5 restriction *in vitro*. (A) Schematic of the chimeric F-MLV constructs expressing the amphotropic envelope. Shown on the right are immunoblots of equal amounts of virions produced in 293T cells transfected with the different chimeric F-MLV constructs and probed with anti-MuLV antibodies for the detection of gp70, p30, and p15E. (B) Mouse SERINC3 and mouse SERINC5 incorporation into the budding virions is dependent on glyco-Gag and not on the viral envelope. 293T cells were cotransfected with F-MLV/Ampho^Env^, gGag^−^F-MLV/Ampho^Env^ (left panel), and gGag^mut^F-MLV/Ampho^Env^ (right panel) and mSERINC3 or mSERINC5 or empty vector as indicated. At 48 h posttransfection, cells and released virus in the culture media were harvested and analyzed by immunoblotting using anti-MLV p30, anti-HA (for detection of mSERINC3 and mSERINC5), and anti-β-actin antibodies. (C and D) Mutations in glyco-Gag result in loss of infectivity in the chimeric viruses. NIH 3T3 cells were infected with F-MLV/Ampho^Env^, gGag^−^F-MLV/Ampho^Env^, and gGag^mut^F-MLV/Ampho^Env^ (MOI 0.1). Virus replication was monitored in the culture supernatants by MLV p30 CA ELISAs at the indicated time points (C) and in the infected cells by isolating genomic DNA and determining MLV DNA levels by RT-qPCR followed by normalization to GAPDH (D). (E to G) mSERINC3 and mSERINC5 restrict virions with amphotropic envelope *in vitro*. Mus dunni cells were infected with equal amounts of 293T-derived F-MLV/Ampho^Env^ (E), gGag^mut^F-MLV/Ampho^Env^ (F), and gGag^−^F-MLV/Ampho^Env^ (G) produced in the presence of mSERINC3 or mSERINC5 or empty vector. Cells were harvested 5 h postinfection, and MLV DNA levels were measured by RT-qPCR and normalized to GAPDH. The percentage (%) of relative infectivity was determined with respect to virus generated in the presence of empty vector. All results are presented as means ± SD. Statistical significance was determined by unpaired (two-tailed) *t* test for data points at 5 dpi (C and D) and by one-way ANOVA and Tukey’s test (E to G). *, *P* < 0.05; **, *P* < 0.01; ns, not significant. Results are shown for *n* = 3 independent experiments (A to G); representative immunoblotting results are shown in panel A and B. mS3, mouse SERINC3; mS5, mouse SERINC5; E.V., empty vector.

It was previously shown that envelopes from different HIV subtypes were able to overcome SERINC5 restriction without preventing SERINC5 incorporation into the virions (35, 36). Therefore, virus susceptibility to human SERINC3 and SERINC5 restriction is dependent not only on glyco-Gag but also on the viral envelope (2–5, 35). We thus hypothesized that the envelope of MLV might play a crucial role in mSERINC5-mediated restriction *in vivo*. The amphotropic MLV envelope, just like the xenotropic MLV envelope, rendered MLV pseudoviruses sensitive to SERINC5 restriction (5, 33), and the glyco-Gag sequences of amphotropic MLV and ecotropic MLV are 90% identical (data not shown). We developed chimeric F-MLV constructs with the aforementioned mutations in glyco-Gag, but the envelope was replaced with that of amphotropic MLV strain 4070A (Fig. 5A)—gGag^−^F-MLV/Ampho^Env^ and gGag^mut^F-MLV/Ampho^Env^. As a control, we also replaced the envelope of F-MLV WT with that of amphotropic 4070A-F-MLV/Ampho^Env^ (Fig. 5A). To verify similar levels of incorporation of the amphotropic MLV envelope glycoproteins into the nascent virions generated by the three chimeric constructs, we performed Western blotting on purified virions and probed for gp70 and p15E, the two subunits of the MLV envelope glycoprotein. We found that the three chimeric viruses had similar levels of amphotropic MLV envelope glycoproteins on their virions (Fig. 5A, right panel). To determine whether glyco-Gag still affected mSERINC3 and mSERINC5 incorporation into virions with the amphotropic envelope, we cotransfected 293T cells with the chimeric envelope viral constructs and with either mSERINC3 or mSERINC5. Western blotting of purified virions showed that mSERINC3 and mSERINC5 were incorporated in a glyco-Gag-dependent manner, whereas the virions where glyco-Gag was either absent or mutated (gGag^−^F-MLV/Ampho^Env^ and gGag^mut^F-MLV/Ampho^Env^) had higher mSERINC3 and mSERINC5 levels packaged than the virions with intact glyco-Gag (F-MLV/Ampho^Env^) (Fig. 5B). Thus, we concluded that glyco-Gag blocks the incorporation of mSERINC3 and mSERINC5 into the viral particles independently of the virus envelope.

We then performed growth curve analyses of the different chimeric viruses that we had generated. NIH 3T3 cells were infected at MOI of 0.1, and virus levels were determined at various times postinfection by p30-CA ELISA and by RT-qPCR measuring MLV DNA levels in the infected cells. Remarkably, in contrast to what we saw with the viruses that had ecotropic MLV envelope, the glyco-Gag mutant chimeric viruses carrying the amphotropic MLV envelope (gGag^−^F-MLV/Ampho^Env^ and gGag^mut^F-MLV/Ampho^Env^) replicated at considerably lower levels than the chimeric virus carrying an intact glyco-Gag (F-MLV/Ampho^Env^) (Fig. 5C and D). Therefore, due to the different growth kinetics of the three viruses (gGag^−^F-MLV/Ampho^Env^, gGag^mut^F-MLV/Ampho^Env^, and F-MLV/Ampho^Env^), we have abstained from making any direct comparisons among the different viruses. To examine the effect of mSERINC3 and mSERINC5 on the infectivity of the chimeric viruses, we infected Mus dunni cells with equal amounts of the three chimeric viruses (gGag^−^F-MLV/Ampho^Env^, gGag^mut^F-MLV/Ampho^Env^, and F-MLV/Ampho^Env^) that were generated in the presence of either mSERINC3 or mSERINC5. We then measured MLV DNA levels by RT-qPCR and found that the glyco-Gag-expressing chimeric virus, F-MLV/Ampho^Env^, replicated at the same levels in infected Mus dunni cells regardless of the presence of mSERINC3 or mSERINC5 (Fig. 5E). On the other hand, the chimeric viruses in which glyco-Gag was either mutated or deleted (gGag^mut^F-MLV/Ampho^Env^ and gGag^−^F-MLV/Ampho^Env^) replicated very poorly in the presence of mSERINC5 (Fig. 5F and G). Interestingly, incorporation of mSERINC3 into the chimeric virions, even when glyco-Gag was disrupted, had no effect on virus infectivity (Fig. 5F and G). This is particularly interesting as the human homologue of mSERINC3, human SERINC3, has antiretroviral functions (3, 4, 37). Thus, we concluded that mSERINC5, similarly to human SERINC5, restricts retrovirus infection *in vitro* in a glyco-Gag-dependent and envelope-dependent manner whereas mSERINC3 has no antiretroviral function *in vitro*.

### Murine SERINC5 restricts MLV infection *in vivo* in a glyco-Gag-dependent and envelope-dependent manner.

**FIG 6:**
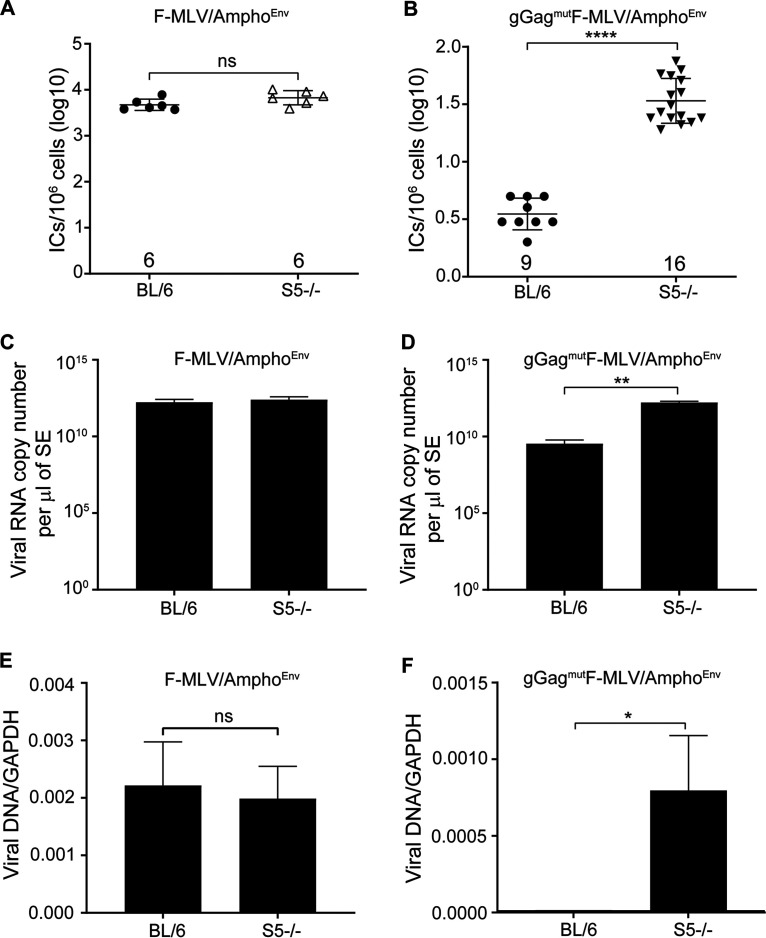
mSERINC5 restricts MLV infection *in vivo* in an envelope-dependent and glyco-Gag-dependent manner. (A and B) Newborn mice were infected with F-MLV/Ampho^Env^ (A) and gGag^mut^F-MLV/Ampho^Env^ (B). Virus titers in spleens were measured 10 days postinfection. Each point represents the titer obtained from an individual mouse, and the mean for each group is shown by a horizontal line. Mice were derived from 4 to 6 litters each; the C57BL/6 and SERINC5^−/−^ mice represent littermates. Numbers of mice used in each group are indicated in the *x* axis. (C and D) Newborn mice were infected with F-MLV/Ampho^Env^ (C) or with gGag^mut^F-MLV/Ampho^Env^ (D), and splenic extracts were prepared in 5 ml (for panel C) or 2 ml (for panel D) of RPMI media 16 days postinfection. Viral RNA copy numbers in the splenic extracts were determined using RT-qPCR. For panel C, *n* = 4 C57BL/6 mice and *n* = 8 SERINC5^−/−^ mice; for panel D, *n* = 3 C57BL/6 mice and *n* = 3 SERINC5^−/−^ mice. (E and F) Equal amounts of viral RNA copies (from the experiments described in the panel C and D legends) were used to infect Mus dunni cells. Genomic DNA was isolated 19 h postinfection, viral DNA levels were measured by RT-qPCR and normalized to GAPDH, and results are presented. All results are presented as means ± SD. Statistical significance was determined by Mann-Whitney (two-tailed) test (A and B) and by unpaired *t* test (C to F). *, *P* < 0.05; **, *P* < 0.01; ****, *P* < 0.0001; ns, not significant. S5, SERINC5; BL/6, C57BL/6; SE, splenic extracts; ICs, infectious centers.

The change in the susceptibility to mSERINC5 restriction seen upon replacing the envelope of the ecotropic F-MLV with that of the amphotropic MLV, 4070A, provides further proof that the envelope of the viral particles is critical for SERINC5 restriction. To examine if the viral envelope affects SERINC5 restriction *in vivo*, we used the chimeric viruses F-MLV/Ampho^Env^, gGag^−^F-MLV/Ampho^Env^, and gGag^mut^F-MLV/Ampho^Env^ to infect SERINC5^−/−^ and BL/6 mice. Titers in the spleens of the infected mice were measured 10 dpi. In the case of F-MLV/Ampho^Env^, the SERINC5^−/−^ and BL/6 mice had similar viral titers in their spleens, which is attributed to the fact that the virus has an intact glyco-Gag (Fig. 6A). Interestingly, in the case of the gGag^−^F-MLV/Ampho^Env^, infection was abrogated for all animals independently of genotype (SERINC5^−/−^, APOBEC3^−/−^, and BL/6 mice) (Fig. S4). This was quite surprising, as in the context of the ecotropic envelope, deletion of glyco-Gag only resulted in reduced virus titers in the spleens of the infected BL/6 and SERINC5^−/−^ mice (Fig. 4) and not in abrogation of infection such as we saw with the amphotropic-envelope-containing chimeric virus. Remarkably, in the case of gGag^mut^F-MLV/Ampho^Env^, we observed that the virus titers in the spleens of the SERINC5^−/−^ mice were significantly higher than those observed in the BL/6 mice (Fig. 6B). This finding not only demonstrates that mSERINC5 restricts MLV infection *in vivo* but also that MLV susceptibility to SERINC5 inhibition is dependent on both the viral envelope and the presence of glyco-Gag.

10.1128/mBio.00588-20.4 FIG S4 Glyco-Gag-deleted amphotropic MLV does not replicate *in vivo.* Newborn mice were infected with gGag^−^F-MLV/Ampho^Env^, and virus titers in spleens were measured 10 days postinfection. Each point represents the titer obtained from an individual mouse, and the mean for each group is shown by a horizontal line. Mice were derived from 3 or 4 litters each; the C57BL/6 and SERINC5^−/−^ data represent the littermates. Numbers of mice used in each group are indicated in the *x* axis. (S5, SERINC5; BL/6, C57BL/6; A3^−/−^, APOBEC3^−/−^; ICs, infectious centers) Download FIG S4, TIF file, 0.4 MB. Copyright © 2020 Timilsina et al. 2020 Timilsina et al. This content is distributed under the terms of the Creative Commons Attribution 4.0 International license.

Our *in vitro* data show that glyco-Gag mutant viral particles with the amphotropic envelope (gGag^mut^F-MLV/Ampho^Env^) have decreased infectivity when mSERINC5 is packaged in the virions. The spleen is one of the major organs that becomes infected by MLV *in vivo* (11, 38–41) Thus, viral particles purified from the spleens of infected BL/6 mice should have reduced infectivity compared to those derived from SERINC5^−/−^ infected mice. To test this, we purified virions from the spleens of BL/6 and SERINC5^−/−^ mice infected with either gGag^mut^F-MLV/Ampho^Env^ or F-MLV/Ampho^Env^. We initially measured viral RNA levels in the splenic extracts and found that F-MLV/Ampho^Env^ viral RNA levels were similar in BL/6 and SERINC5^−/−^ mice (Fig. 6C) whereas gGag^mut^F-MLV/Ampho^Env^ viral RNA levels were higher in the splenic extracts from the SERINC5^−/−^ mice than in those from the BL/6 mice (Fig. 6D), which reflects the higher infection levels of the former (Fig. 6B). Subsequently, we infected Mus dunni cells with equal amounts of gGag^mut^F-MLV/Ampho^Env^ or F-MLV/Ampho^Env^ viral particles isolated from BL/6 or SERINC5^−/−^ mice after normalizing to viral RNA levels. We measured MLV DNA levels by RT-qPCR and found no difference in the levels of infectivity of F-MLV/Ampho^Env^ virions isolated from either BL/6 or SERINC5^−/−^ mice (Fig. 6E). On the other hand, gGag^mut^F-MLV/Ampho^Env^ virions isolated from SERINC5^−/−^ mice were more infectious than those isolated from BL/6 mice (Fig. 6F). The absence of an antibody that could detect endogenous levels of mSERINC5 prevented us from examining mSERINC5 incorporation into the nascent virions *in vivo*. Taken together, these data suggest that mSERINC5 is packaged into virions isolated from the spleens of infected mice in a glyco-Gag-dependent manner and limits viral infectivity.

### SERINC5 and APOBEC3 additively act to restrict retrovirus infection *in vivo*.

**FIG 7:**
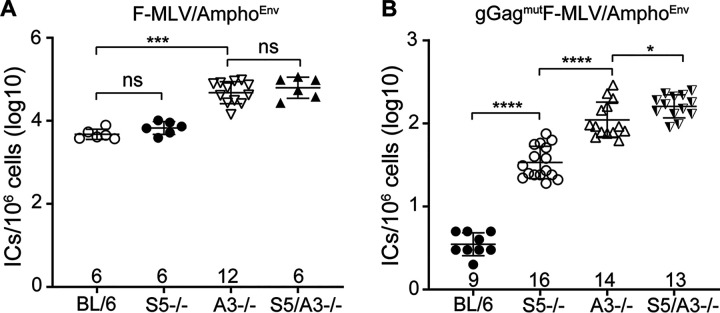
SERINC5 and APOBEC3 have additive effect on MLV infection *in vivo.* Newborn mice were infected with F-MLV/Ampho^Env^ (A) and gGag^mut^F-MLV/Ampho^Env^ (B). Virus titers in the spleens were measured 10 days postinfection. Each point represents the titer obtained from an individual mouse, and the mean for each group is shown by a horizontal line. Mice were derived from 2 to 6 litters each; the BL/6 and SERINC5 mice represent littermates. Numbers of mice used in each group are indicated on the *x* axis. All results are presented as means ± SD. Virus titers for BL/6 and SERINC5^−/−^ mice shown in panels A and B for F-MLV/Ampho^Env^ and gGag^mut^F-MLV/Ampho^Env^ are duplicated from Fig. 6A and B, respectively. Statistical significance was determined by Mann-Whitney (two-tailed) test. *, *P* < 0.5; ***, *P* = 0.0001; ****, *P* < 0.0001; ns, not significant. S5, SERINC5; BL/6, C57BL/6; A3, APOBEC3.

Mouse APOBEC3 potently restricts MLV infection *in vivo* by blocking reverse transcription (11, 22, 24, 38, 41, 42). SERINC5 blocks virus-cell fusion (7, 8). As mouse APOBEC3 and mSERINC5 target different steps of the retrovirus life cycle and both restrict MLV infection *in vivo*, we asked whether these two factors act additively to block retrovirus infection *in vivo*. To examine any additive effect between mouse APOBEC3 and mSERINC5, we developed double knockout mice for these two genes, resulting in APOBEC3/SERINC5^−/−^ mice. We infected APOBEC3/SERINC5^−/−^ mice with F-MLV/Ampho^Env^ and gGag^mut^F-MLV/Ampho^Env^ and harvested their spleens at 10 dpi to determine viral titers. F-MLV/Ampho^Env^ replicated at similar levels in the spleens of APOBEC3^−/−^ and APOBEC3/SERINC5^−/−^ mice (Fig. 7A). In the case of gGag^mut^F-MLV/Ampho^Env^, the APOBEC3/SERINC5^−/−^ mice had higher viral titers in their spleens than either the APOBEC3^−/−^ mice or the SERINC5^−/−^ mice (Fig. 7B). Therefore, we concluded that mouse APOBEC3 and mSERINC5 act additively to block retrovirus infection *in vivo*.

### Murine SERINC3 has no effect on MLV restriction *in vivo*.

**FIG 8:**
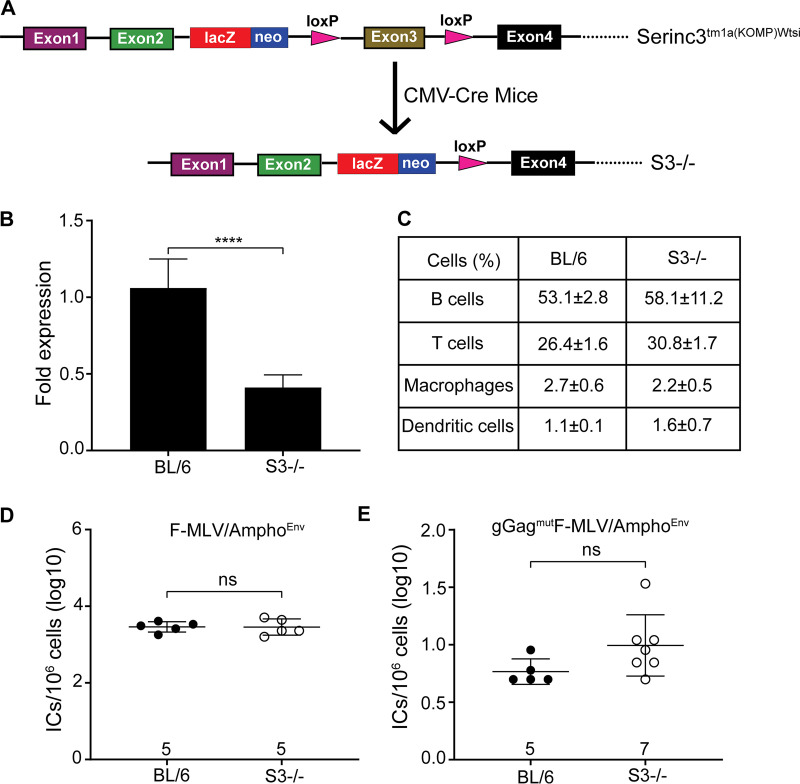
mSERINC3 has no effect on MLV infection *in vivo*. (A) Schematic of the derivation of SERINC3^−/−^ mice. Serinc3^tm1a(KOMP)Wtsi^ mice provided by the Wellcome Trust Sanger Institute were crossed with the Cre-Deleter mice (Taconic Biosciences), leading to the loss of exon 3 and a frameshift mutation abrogating the SERINC3 gene. (B) Total RNA was isolated from C57BL/6 (*n* = 9) and SERINC3^−/−^ (*n* = 9) mice, and SERINC3 levels were measured by RT-qPCR and normalized to GAPDH. SERINC3 transcript fold expression was determined relative to that in C57BL/6 mice. (C) PBMCs from 3 C57BL6 mice and 4 SERINC3^−/−^ mice were stained with anti-CD45R/B220 (B cells), anti-CD3 (T cells), anti-F4/80 (Macrophages), and anti-CD11c (Dendritic cells) and subjected to FACS analysis. Values are presented as means ± SD. There were no significant differences in the percentages of T cells, B cells, macrophages, and dendritic cells among the C57BL/6 and SERINC3^−/−^ mice. (D and E) C57BL/6 and SERINC3^−/−^ newborn mice were infected with F-MLV/Ampho^Env^ (D) and gGag^mut^F-MLV/Ampho^Env^ (E). Virus titers in spleens were measured 10 days postinfection. Each point represents the titer obtained from an individual mouse, and the mean for each group is shown by a horizontal line. Mice were derived from 2 to 4 litters each. Numbers of mice used in each group are indicated on the *x* axis. All results are presented as means ± SD. Statistical significance was determined by unpaired *t* test (B and C) and by Mann-Whitney (two-tailed) test (D and E). ****, *P* < 0.0001; ns, not significant. S3, SERINC3; BL/6, C57BL/6; ICs, infectious centers.

SERINC3 is another member of the SERINC protein family that has antiretroviral functions (3, 4). To determine whether mSERINC3 could exert an antiretroviral function *in vivo*, we acquired mice from the Wellcome Trust Sanger Institute Mouse Genetics Project with loxP sites flanking exon 3 of SERINC3. We crossed these mice with CMV-Cre-expressing mice (Cre Deleter; Taconic) to develop complete “−/−” mice for mSERINC3 (Fig. 8A), as deletion of exon 3 results in a truncated mSERINC3 RNA transcript with a frameshift mutation that leads to a premature stop codon. Because there is no antibody to detect endogenous mSERINC3 protein levels, we probed for mSERINC3 mRNA levels by RT-qPCR and found that the SERINC3^−/−^ mice had significantly lower levels of mSERINC3 transcripts than the BL/6 mice (Fig. 8B). The SERINC3^−/−^ mice had no apparent physical or behavioral deficits and had leukocyte populations similar to those seen with the BL/6 mice (Fig. 8C). Subsequently, we infected SERINC3^−/−^ mice with either gGag^mut^F-MLV/Ampho^Env^ or F-MLV/Ampho^Env^. At 10 dpi, the two viruses replicated the same in the spleens of either BL/6 or mSERINC3^−/−^ mice (Fig. 8D and E). Thus, mSERINC3, unlike human SERINC3, has no effect on MLV infection *in vivo*.

### MLV sensitivity to mSERINC5 is independent of the route of virus entry into target cells.

**FIG 9:**
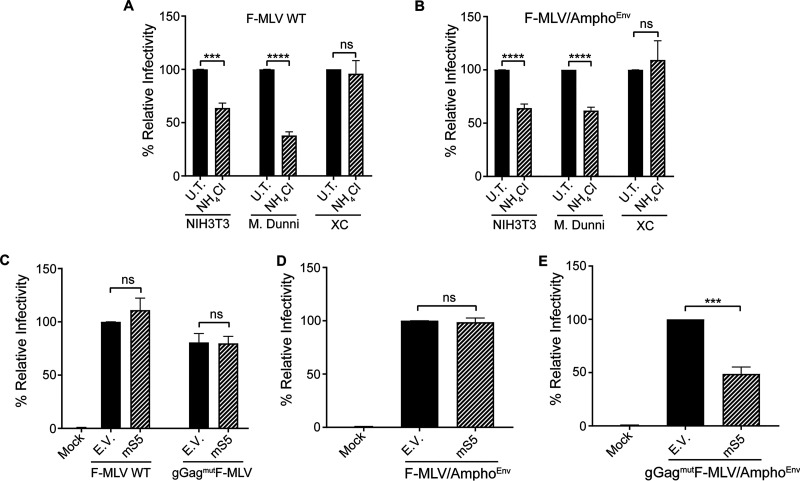
MLV sensitivity to mSERINC5 restriction is independent of the route of virus entry. (A and B) NIH 3T3, Mus dunni, and XC cells were infected with F-MLV WT (A) and F-MLV/Ampho^Env^ (B) (MOI 0.5) in the presence or absence of 30 mM ammonium chloride (NH_4_Cl). Cells were harvested 19 h postinfection, and MLV DNA levels were measured by RT-qPCR and normalized to GAPDH. The percentage (%) of relative infectivity was determined with respect to the untreated control (U.T.). (C to E) XC cells were infected with equal amounts of F-MLV WT and gGag^mut^F-MLV (C), F-MLV/Ampho^Env^ (D), and gGag^mut^F-MLV/Ampho^Env^ (E) produced in the presence of mSERINC5 or empty vector. Cells were harvested 5 h postinfection, and MLV DNA levels were measured by RT-qPCR and normalized to GAPDH. The percentage (%) of relative infectivity was determined with respect to virus generated in the presence of empty vector. All results are presented as means ± SD. Statistical significance was determined by unpaired (two-tailed) *t* test. ***, *P* < 0.001; ****, *P* < 0.0001; ns, not significant. Results are shown for *n* = 3 independent experiments. NH_4_Cl, ammonium chloride; U.T., untreated; mS5, mouse SERINC5; E.V., empty vector.

Previous studies on the envelope of ecotropic MLVs such as Moloney MLV (MoMLV) and F-MLV showed that virus internalization varies with the host cell (43–45). Our studies showed that infection of NIH 3T3 and Mus dunni cells, both murine fibroblasts, was affected by the presence of ammonium chloride, a pH inhibitor (Fig. 9A), which, in agreement with previous reports, suggested that virus fusion takes place in a low-pH environment after receptor-mediated endocytosis (44–46). On the other hand, in accord with previous reports (44–49), ecotropic MLV infection is pH independent in XC cells, a rat sarcoma cell line, as we did not observe any effect on virus infection when ammonium chloride was added to the cells (Fig. 9A). In the case of amphotropic MLV, fusion happens on the cell surface and occurs at extracellular neutral pH (45, 48). However, another study found that entry of amphotropic MLV into NIH 3T3 cells was pH dependent (47). Similarly to that report, we found that treatment of NIH 3T3 cells and Mus dunni with ammonium chloride blocked both ecotropic and amphotropic MLV infection (Fig. 9A and B). To rule out whether the route of entry of ecotropic MLVs is responsible for the resistance of F-MLV to mSERINC5 restriction, we infected XC cells (where entry occurs by surface fusion and is pH independent) with equal amounts of F-MLV WT, gGag^mut^F-MLV, gGag^mut^F-MLV/Ampho^Env^, or F-MLV/Ampho^Env^ generated in the presence of either mSERINC5 or empty vector. XC cells were infected with equal amounts of virus, DNA was isolated 5 h postinfection (hpi), and RT-qPCR was performed to measure MLV DNA levels. As expected, F-MLV WT and F-MLV/Ampho^Env^ had similar MLV DNA levels in the infected XC cells independently of the presence of mSERINC5 (Fig. 9C and D). In the case of gGag^mut^F-MLV, virus DNA levels were similar in XC cells regardless of the presence of mSERINC5 in the virions (Fig. 9C), similarly to what we observed when we infected Mus dunni cells (Fig. 3E). However, when we infected XC cells with gGag^mut^F-MLV/Ampho^Env^, we observed that viral DNA levels were reduced when the virus was produced in the presence of mSERINC5 (Fig. 9E). These results are similar to what we had observed before when we used Mus dunni cells (Fig. 5F). Thus, the findings described above suggest that, as previously hypothesized, the resistance of ecotropic MLV to mSERINC5 is independent of the route of virus entry.

## DISCUSSION

*In vitro* studies have shown that SERINC5 can block retrovirus infection (3–5, 25). However, many retroviruses have developed mechanisms that can counteract SERINC5-mediated restriction. In the case of MLV, glyco-Gag inhibits SERINC5 restriction by removing SERINC5 from the plasma membrane and thus blocking its incorporation into the budding virions (25, 31). While much work has been done to understand the *in vitro* antiretroviral functions of SERINC proteins (3–5, 25, 43), nothing is known about the antiretroviral function of this family of proteins *in vivo*. For many antiretroviral proteins, e.g., SAMHD1, their antiviral effect *in vitro* is not always recapitulated *in vivo* (27). Many times, *in vitro* experiments are done using cell types that are not naturally infected by retroviruses or SERINC proteins are expressed at levels significantly higher than physiological levels. Finally, natural MLV infection in mice involves a multitude of different cell types, something that cannot be easily examined *in vitro*.

In order to examine whether SERINC3 and SERINC5 have antiretroviral properties *in vivo*, we generated SERINC5^−/−^ mice and confirmed the disruption of the gene by RT-qPCR, as there is no antibody available to detect endogenous mSERINC5 protein levels. SERINC5^−/−^ mice had no apparent defects and thus represent a useful model to examine the *in vivo* effect of mSERINC5 on retrovirus infection. Using wild-type and glyco-Gag mutant F-MLV, we saw no differences in viral titers in the spleens of either the SERINC5^−/−^ or the BL/6 infected mice. On the other hand, we observed glyco-Gag-dependent exclusion of mSERINC3 and mSERINC5 from the budding virions. We speculated that the lack of any obvious antiviral effect may have resulted from the fact that the viruses used in these experiments had the ecotropic envelope, which makes them resistant to SERINC5 restriction (5, 43). Previous reports from studies using pseudoviruses showed that the presence of amphotropic or xenotropic MLV envelope on the surface of virions rendered them susceptible to human SERINC5 restriction *in vitro* (5, 33). To examine the effect of the envelope on mSERINC5 restriction in fully infectious virus clones, we generated chimeric F-MLV constructs in which the ecotropic envelope was replaced with that of amphotropic MLV. Using these chimeric viruses, we found that the glyco-Gag mutant MLV with an amphotropic envelope replicated at higher levels in the spleens of SERINC5^−/−^ mice than in those of BL/6 mice, demonstrating the critical role of the viral envelope on mSERINC5 restriction *in vivo*. This report provides evidence that mSERINC5 is an antiretroviral factor *in vivo* and that the retroviral envelope is important in determining *in vivo* sensitivity to mSERINC5 restriction. Of note is the striking difference in the glyco-Gag requirement for virus infectivity between viral particles with the amphotropic envelope and those with ecotropic envelope. While the former required glyco-Gag for infectivity both *in vivo* and *in vitro*, the latter were unaffected *in vitro* whereas there was only about a 10-fold drop in infectivity *in vivo* when glyco-Gag was mutated. It is not clear why the presence of different envelopes modulates the role of glyco-Gag in virus infectivity. The relationship between envelope and glyco-Gag needs to be elucidated. To circumvent the restriction represented by the absence of an antibody that detects endogenous mSERINC5, we compared the levels of infectivity between viral particles with the amphotropic envelope and mutated glyco-Gag isolated from the spleens of either SERINC5^−/−^ or BL/6 infected mice. We found that virions isolated from the SERINC5^−/−^ mice were more infectious than those derived from BL/6 mice, which suggests that mSERINC5 is incorporated into virions *in vivo* and blocks virus infectivity. Furthermore, it has been hypothesized in the field that the route of entry of a virus could affect SERINC5 restriction. Our data suggest that the route of entry of a virus does not affect susceptibility to SERINC5 restriction, as ecotropic MLV was resistant to SERINC5 restriction both in Mus dunni cells, where the virus enters in a pH-dependent manner, and in XC cells, where entry occurs independently of pH. Thus, another factor and not the route of entry of a virus affects the susceptibility of the retrovirus envelope to SERINC5.

Similarly to previously reported characteristics of mouse APOBEC3 (24, 38, 41, 50), our findings show that mSERINC5 potently restricts MLV infection *in vivo*. SERINC5 and mouse APOBEC3 restrict retrovirus infection at different steps of the retroviral life cycle, SERINC5 during virus-cell fusion (7) and APOBEC3 during reverse transcription (42, 50). Using SERINC5/APOBEC3 double knockout mice, we showed that they indeed act additively to block retrovirus infection. To our knowledge, this is the first time that two different antiretroviral factors have been shown to act in unison *in vivo*. However, how these two factors come together to additively restrict MLV infection *in vivo* needs further investigation.

We also examined the role of mSERINC3 in retrovirus infectivity *in vivo* and *in vitro*. Human SERINC3 has been shown to be antiretroviral, albeit it is not as potent as SERINC5 (3, 4), while there are differing opinions on the antiretroviral role of mSERINC3 (25, 51). Our *in vitro* and *in vivo* findings showed that mSERINC3 has no antiretroviral functions against MLV, although it is targeted for internalization by glyco-Gag *in vitro*. It is possible that the antiretroviral function of the human homologue of SERINC3 was acquired at a later point in the evolutionary scale, possibly due to selective pressure from a retrovirus that was absent in the mouse evolutionary history, or it is possible that the murine SERINC3 has viral targets different from those associated with the human homologue.

## MATERIALS AND METHODS

### Ethics statement.

All mice were housed and bred according to the policies of the Institutional Animal Care and Use committee of the University at Buffalo. The experiments performed with mice in this study were approved by this committee (MIC 46038Y).

### Generation of SERINC5^−/−^ mice.

To generate the SERINC5^−/−^ mice, exon1 and exon 2 were targeted by 2 guide RNAs (gRNAs) (gRNA1 [5′-ATGTCTGCCCGGTGCTGTGCTGG] and gRNA2 [5′-GCCCTAAGTTCCGACAGTCTCGG]) by the use of IDT Alt-R crRNA/trRNA CRISPR/Cas9 technology. The gRNAs and recombinant Cas9 (RNPs) were electroporated into zygotes of C57BL/6N mice (Charles River Laboratories). DNA from the mice generated from the electroporations was sent to the Genome Engineering and iPSc Center at Washington University in St. Louis, where it were screened via targeted next-generation sequencing (NGS) for the presence of deletion or indels at the target site. One founder line was discovered to have a sizable 49-kb deletion that included part of exon 1 and exon 2 along with the intron between these two exons. As such, for the purpose of genotyping we used two sets of primers, SEREx1F and SEREx2R/SEREx1F and SERInt2R (see Table S1 in the supplemental material); the first set of primers amplifies the deleted allele (as the two primers are 49 kb apart in the BL/6 mice), and the second set of primers amplifies a region of about 250 bp.

10.1128/mBio.00588-20.5 TABLE S1 Primers used for genotyping the mice. Download Table S1, DOCX file, 0.01 MB. Copyright © 2020 Timilsina et al. 2020 Timilsina et al. This content is distributed under the terms of the Creative Commons Attribution 4.0 International license.

### Generation of SERINC5/APOBEC3^−/−^ mice.

APOBEC3 mice were bred at The State University of New York (SUNY), University at Buffalo, as previously described (11, 52). The SERINC5^−/−^ mice described in this study were crossed with the APOBEC3^−/−^ mice to generate SERINC5/APOBEC3^−/−^ mice.

### Generation of SERINC3^−/−^ mice.

SERINC3^−/−^ mice were derived from *in vitro* fertilization of C57BL/6N females with sperm from a single heterozygous male from the mutant mouse line C57BL/6NTac-SERINC3^tm1a(KOMP)Wtsi^/WtsiFlmg constructed by the Wellcome Trust Sanger Institute Mouse Genetics Project (Sanger MGP). Cre-Deleter mice [C57BL/6NTac-Gt(ROSA)26Sor^tm16(cre)Arte^], where the cre gene is expressed under the control of the Gt(ROSA)26Sor gene, were purchased from Taconic Biosciences and used to develop SERINC3^−/−^ mice. Deletion by cre recombinase in SERINC3^tm1a(KOMP)Wtsi^/WtsiFlmg results in the removal of exon 3, leading to a frameshift mutation and a premature stop codon. The following primers were used for genotyping: Serinc3_35556_F and Serinc3_35556_R/SERINC3Ex3F and SERINC3Ex3R for detection of WT alleles and Serinc3_35556_F and CAS_R1_Term/DelF and DelR for detection of deleted alleles. The primers used for detection of cre were CreF and CreR (see Table S1).

### Cell culture and transfection.

293T cells (ATCC) and 293FT cells (Invitrogen) were cultured in Dulbecco’s modified Eagle media (DMEM; Gibco) with 10% (vol/vol) fetal bovine serum (FBS; Sigma), 0.1 mM nonessential amino acids (Gibco), 6 mM l-glutamine (Gibco), 100 mg/ml penicillin and streptomycin (P/S; Gibco), 1 mM sodium pyruvate (Gibco), and 500 μg/ml Geneticin (Gibco). NIH 3T3 cells and XC cells (ATCC) were cultured in DMEM with 10% FBS and P/S. Mus dunni cells were maintained in RPMI media with 10% FBS and P/S. EL4 cells (ATCC) were cultured in RPMI media with 10% FBS, P/S, and 0.05 mM β-mercaptoethanol (βME; Bio-Rad). MutuDC1940 cells (30) were cultured in Iscove's modified Dulbecco's media (IMDM) with 8% FBS, 100 mg/ml P/S, 1 mM sodium pyruvate, 10 mM HEPES (Corning), and 0.05 mM βME. All transfections were performed using a Lipofectamine 3000 transfection kit (Invitrogen) per the manufacturer’s recommendation.

### Plasmids.

The F-MLV WT provirus construct pLRB302 has been previously described (53, 54). To generate glyco-Gag-deficient F-MLV (gGag^−^F-MLV) provirus, two stop codons were introduced at residues 32 and 55 of the glyco-Gag reading frame using a Phusion SDM kit (Thermo Fisher Scientific). The primers used to introduce the changes (underlined) were as follows: 5′-TGCACCCCCCTAAGAGGAGGGGT-3′/5′-CCAAAGAGTCCAAAACGATCGGGATGG-3′ and 5′-GTCTGAGTTTTAGCTTTCGGTTTGGAA-3′/5′-GGGGGCGGAAACCGTTTTAGCC-3′. To minimize polymerase-driven errors, an XhoI-EcoRI fragment from a gGag^−^F-MLV construct was subcloned into the F-MLV WT backbone. Glyco-Gag-mutated F-MLV (gGag^mut^F-MLV) was generated by mutating P31, Y36, L39, and R63 residues of glyco-Gag to alanine by site-directed mutagenesis using a Phusion SDM kit (Thermo Fisher Scientific) (the primer pairs used were 5′-CTCTTTGGTGCACCCGCCTTAGAGGAGGGGGCTGTGGTTGCGGTAGGAGACAGAGG-3′/5′-TCCAAAACGATCGGGATGGTTGGACTC-3′ for P31A, Y36A, and L39A and 5′-CTTTCGGTTTGGAACCGAAGCGCCGCCGCGCGTCTTGTCTGC-3′/5′-CAAAAACTCAGACGGGGGCGGAAAC-3′ for R63A, where the underlined sequence characters represent the sites of the changed amino acids). A plasmid containing the amphotropic MLV *env* gene construct (pSV-A MLV env) was obtained from NIH AIDS Reagent program, Division of AIDS, NIAID, NIH and SV-A-MLV-env from Nathaniel Landau and Dan Littman (55). F-MLV amphotropic envelope chimeras (F-MLV/Ampho^Env^, gGag^−^F-MLV/Ampho^Env^, and gGag^mut^F-MLV/Ampho^Env^) were created by replacing the entire *env* coding sequence of F-MLV WT with an amphotropic *env* coding sequence from p-SV-A MLV *env* using a NEBuilder HiFi DNA assembly kit (New England Biolabs). The primers used for swapping the *env*-coding regions were as follows: 5′-ATAAAAGATTTTATTTAGTTTCCAGAAAAAGGGGGGA-3′/5′-CGTTGAACGCGCCATGTCGATTCCGATGGTGGCTC-3′ for the F-MLV backbone and 5′-ACCATCGGAATCGACATGGCGCGTTCAACGCTCTC-3′/5′-AATAAAATCTTTTATCTATGGCTCGTACTCTATAGG-3′ for the amphotropic MLV *env*. pBJ5-hSERINC3-HA (pBJ5-hSERINC3-hemagglutinin) and pBJ5-hSERINC5-HA were obtained from Heinrich Gottlinger (4). pBJ5-mSERINC3-HA and pBJ5-mSERINC3-HA were generated by replacing the human SERINC genes with the murine ones. mSERINC3 and mSERINC5 were PCR amplified from mSERINC3 and mSERINC5 cDNAs (Dharmacon Incorportated) using the primers 5′-GGGCTCGAGATGGGGGCCGTCCTCG-3′/5′-CCCGCGGCCGCTTAAGCGTAATCTGGAACATCGTATGGGTAGCTGAAGTCCCGACCTGTG-3′ for mSERINC3 and the primers 5′-GGGCTCGAGATGTCTGCCCGGTGCTGTG-3′/5′-CCCGCGGCCGCTTAAGCGTAATCTGGAACATCGTATGGGTACACAGAGAACTGCCTGGA-3′ for mSERINC5 followed by XhoI/NotI digestion and ligation in the pBJ5 vector. All constructs generated were confirmed by DNA sequencing. Primers used for sequencing are listed in Table S2.

10.1128/mBio.00588-20.6 TABLE S2 Primers used for sequencing of proviral constructs. Download Table S2, DOCX file, 0.01 MB. Copyright © 2020 Timilsina et al. 2020 Timilsina et al. This content is distributed under the terms of the Creative Commons Attribution 4.0 International license.

### Virus preparation for animal experiments.

Virus stocks were prepared by transfecting 293FT cells (Invitrogen) seeded in 10-cm-diameter cell culture dishes with the viral constructs described above. Culture supernatants were harvested 48 h after transfection, filtered, and treated with 10 U/ml DNase I (Roche) for 40 min at 37°C. Titers of viruses were determined by infecting Mus dunni cells for 48 h. For the ecotropic MLV viruses, immunofluorescent focus assays (IFAs) were performed to count infectious centers (ICs) using Mab720 (56) followed by DAB (3,3′-diaminobenzidine) staining with horseradish peroxidase (HRP) substrate (Vector Laboratories). In the case of the chimeric viruses with the amphotropic MLV envelope, IFAs were performed using Mab573 (57) followed by immunostaining with Alexa Fluor 488-conjugated goat anti-mouse antibody (Invitrogen); samples were then processed using a BioTek Cytation 1 imaging plate reader (BioTek).

### Detection of incorporation of SERINC3/5 into virions by immunoblotting.

A total of 0.5 × 10^6^ 293T cells were seeded in 6-well culture plates and cotransfected with viral DNA construct (5 μg) and pBJ5-mSERINC3-HA or pBJ5-mSERINC5-HA or empty vector (100 ng). Cells and culture media were harvested 48 h after transfection. Cell lysates were prepared as previously described (6). Briefly, cells were lysed in DM lysis buffer (0.5% [wt/vol] n-decyl-β-d-maltopyranoside, 20 mM Tris-HCl [pH 7.5], 10% [vol/vol] glycerol, 1× protease inhibitors, 25 U/ml Benzonase] and were centrifuged at 14,000 rpm followed by the collection of the supernatant fraction. Culture media were pelleted through a 30% sucrose cushion as previously described (11). Cell lysates or virus pellets were mixed with 1× sample loading buffer and incubated at 37°C for 15 min before they were resolved on 10% sodium dodecyl sulfate polyacrylamide gels. Blots were probed using the following antibodies: polyclonal goat anti-MLV (11) (NCI repository), rat anti-MLV p30 R187 (ATCC CRL-1912), rat anti-MuLV 83A25 (58), rat anti-MuLV transmembrane protein/p15E (Kerafast), rabbit anti-HA (Cell Signaling Technology), monoclonal anti-β-actin (Sigma-Aldrich). HRP-conjugated anti-rabbit (Cell Signaling Technology), HRP-conjugated anti-rat (Cell Signaling Technology), HRP-conjugated anti-mouse (EMD Millipore), and HRP-conjugated anti-goat (Sigma-Aldrich) were used for detection using the enhanced chemiluminescence detection kits Clarity and Clarity Max ECL (Bio-Rad).

### Target cell assays.

Virions were prepared and treated with 10 U/ml DNase I (Roche) for 40 min at 37°C as described above. Virus preparations were normalized following quantitation by MLV-p30 immunoblotting and analysis using ImageJ software (National Institutes of Health; https://imagej.nih.gov/ij/). Equal amounts of viruses were used to infect 0.8 × 10^5^ Mus dunni or XC sarcoma cells in a 6-well plate. Cells were harvested 5 hpi. DNA was extracted using a DNeasy blood and tissue kit (Qiagen) per manufacturer’s instructions, and RT-qPCR was performed using a Power SYBR green PCR master mix kit (Applied Biosystems). The MLV primers used were employed for the ecotropic MLV infections (5′-TACAGGGAGCTTACCAGGCA-3′/5′-GTTCCTATGCAGAGTCCCCG-3′) and for the amphotropic envelope chimeric MLV infections (5′-AAGTCCAAGTGTCCCACAGC-3′/5′-AGCCCACATTGTTCCGGC-3′). GAPDH (glyceraldehyde-3-phosphate dehydrogenase) primers (5′-CCCCTTCATTGACCTCAACTACA-3′/5′-CGCTCCTGGAGGATGGTGAT-3′) were used for normalization. A CFX384 Touch real-time PCR detection system (Bio-Rad) was used for all RT-qPCR assays described in this study. The reactions were performed under the following conditions: 95°C for 10 min, followed by 40 cycles of 95°C for 10 s and 60°C for 30 s. The relative levels of amplification were quantified for each sample from standard curves generated using known quantities of DNA standard templates.

### *In vivo* infections.

For all SERINC5 and SERINC3 *in vivo* experiments, newborn mice were infected by intraperitoneal (i.p.) injections with 5 × 10^3^ ICs of virus as previously described (11, 24, 38). Spleens from infected mice were harvested 10 days postinfection and used for the infectivity assays described below.

### Infectivity assays.

MLV infection levels in the spleens were measured by IC assays as previously described (38). Briefly, Mus dunni cells were seeded in a 6-well plate at a density of 0.8 × 10^5^ per well. The next day, the cells were cocultured with 10-fold dilutions of splenocytes isolated from infected mice. IC levels were determined 48 hpi after performing IFAs as described above in the “Virus preparation for animal experiments” paragraph.

### Growth curve experiment.

NIH 3T3 cells (40,000/well) were seeded in a 6-well plate. Cells were infected the next day with the different viruses used in this project (0.1 MOI). After 2 h of infection, cells were washed 3 times with phosphate-buffered saline (PBS) and cultured in 2 ml media for 5 days. Culture supernatants and cells were harvested each day and stored at –80°C. Concentrations of MLV p30 in the supernatants were determined using a QuickTiter MuLV core antigen ELISA kit (MuLV p30; Cell Biolabs). DNA was isolated from the infected cells using a DNeasy blood and tissue kit (Qiagen), and viral DNA levels were determined by RT-qPCR, as described above (see the “Target cell assays” paragraph).

### SERINC3/5 transcription levels.

Tissues were harvested from 3-month-old BL/6, SERINC5^−/−^, and SERINC3^−/−^ mice, and total RNA was isolated using TRIzol (Invitrogen) per the manufacturer’s instruction. cDNA was synthesized using a SuperScript III first-strand synthesis kit (Invitrogen). RT-qPCR was performed as described above (see “Target cell assays”). The primers used were as follows: for mSERINC3, 5′-TGACCAATGAACCTGAGCGG-3′/5′-GCTGTTGCTCGAAGTACGGA-3′, and for mSERINC5, 5′-GTCCAGAATCGACAGCCACA-3′/5-ATCCGAGCTCGACCTTGTTG-3′. Actin primers (5′-TGGAATCCTGTGGCATCCATGAAAC-3′/5′-TAAAACGCAGCTCAGTAACAGTCCG-3′) were used for normalization.

MutuDC1940 and EL4 cells were infected with MLV by spinoculation as previously described (38). Briefly, cells were seeded in a 96-well plate at a cell density of 0.8 × 10^5^/100 μl of culture media. The next day, the cells were infected with MLV (0.1 MOI) and the plate was centrifuged at 1,800 rpm for 2 h at room temperature. After centrifugation, the cells were washed 3 times with PBS and maintained in 100 μl of media at 37°C. Cells were harvested at different time points. RNA was isolated, and SERINC3/5 expression levels were determined by RT-qPCR as described above (see “Target cell assays”).

### FACS analysis and sorting.

Blood was collected via retro-orbital bleeding from SERINC5^−/−^, SERINC3^−/−^, and BL/6 mice of similar ages. PBMCs were stained with anti-CD3-APC (anti-CD3-allophycocyanin) (BD Biosciences), anti-CD45R/B220-FITC (anti-CD45R/B220-fluorescein isothiocyanate) (BD Biosciences), anti-CD11c-APC (BD Biosciences), and anti-mouse F4/80-FITC (BioLegend) (23). Stained cells were processed using BD LSRFortessa. Results were analyzed using FlowJo software.

B cells, T cells, and DCs were sorted from blood collected via retro-orbital bleeding from adult BL/6 mice (6 weeks old) or via cardiac puncture of neonates (4 days old) using a BD FACSAria fusion cell sorter. Total RNA was isolated using an RNeasy minikit or an RNeasy Plus Micro kit (Qiagen) per the manufacturer’s instruction. SERINC1/2/3/4/5 expression profiles were determined by RT-qPCR as described above (see “Target cell assays”). In addition to the SERINC3/5 and GAPDH primers mentioned above (see “SERINC3/5 transcription levels”), the following primers were used to detect the other SERINC family members: for SERINC1, 5′-CTGTTCAGTGGTGGCATCCTCA-3′/5′-CCTGACTGTTGTTGGAGGTACG-3′; for SERINC2, 5′-AATCAGCGGTGGCTGTGTAAGG-3′/5′-ACATCAGTGCCACAGCAGCGAT-3′; for SERINC4, 5′-CCGCAAGCAACCAAACTCTG-3′/5′-CTGCTACTGAGGTATCCGGTATT-3′.

### Infectivity measurement of *in vivo-*derived virus.

Two-day-old SERINC5^−/−^ and BL/6 mice were infected by i.p. injections with 4 × 10^5^ ICs of virus, and spleens were harvested 16 days postinfection. Spleen extracts were prepared as previously described (59) with some modifications. Briefly, spleens were mashed through a cell strainer and splenocytes were collected in a mixture of 5 ml (for F-MLV/Ampho^Env^ virus) or 2 ml (for gGag^mut^F-MLV/Ampho^Env^ virus) of RPMI media with 10% FBS and 100 mg/ml P/S. Cells were pelleted at 1,200 rpm for 10 min at 4°C. The supernatant was removed and centrifuged at 3,000 rpm for 10 min at 4°C and then filtered through a 0.45-μm-pore-size syringe filter. RNA was isolated from 100 μl of the supernatant using an RNeasy kit (Qiagen) per the manufacturer’s recommendation. cDNA synthesis and RT-qPCR were performed as described above (see “SERINC3/5 transcription levels”). Normalized spleen extracts (based on RNA copies) were used to infect 0.3 × 10^5^ Mus dunni cells seeded in a 24-well plate. Cells were harvested 19 hpi, and the amount of viral DNA in the infected cells was determined by RT-qPCR, as described above (see “Target cell assays”).

### Ammonium chloride treatment.

One day prior to infection, NIH 3T3, Mus dunni, or XC cells were seeded in a six-well plate (0.8 × 10^5^ cells/well). Cells were pretreated with 30 mM ammonium chloride (NH_4_Cl) for 30 min before infection was performed with F-MLV WT or F-MLV/Ampho^Env^ viruses (MOI 0.5) (45). The same concentration of NH_4_Cl was maintained during the infection period and an additional 2 h, after which cells were maintained in fresh media without NH_4_Cl. Cells were harvested 19 hpi, and the amount of viral DNA in the infected cells was determined by RT-qPCR, as described above (see “Target cell assays”).

### Poly(I·C) treatment.

BL/6 mice (3 to 4 weeks old) were injected i.p. with high-molecular-weight poly(I·C) (InvivoGen) (8 mg/kg of body weight) or with PBS as previously described (60). Spleens were harvested 6 h posttreatment. RNA was isolated from the spleens, and SERINC expression levels were determined by RT-PCR as described above (see paragraphs labeled “Target cell assays,” “SERINC3/5 transcription levels,” and “FACS analysis and sorting”). Interferon-stimulated gene 15 (ISG15) served as a positive control, and the primers used for ISG15 detection were 5′-TGACGCAGACTGTAGACACG-3′ and 5′-TGGGGCTTTAGGCCATACTC-3′.

### Statistical analysis.

Statistical analyses were performed using GraphPad Prism software version 8.2. The statistical tests used to determine significance are described in the figure legends. A difference was considered to be significant for *P* values of <0.05.
