# Supplementary figures and images for: SERINC5 Potently Restricts Retrovirus Infection In Vivo
Source: mBio. 2020 Jul 14;11(4):e00588-20. doi: 10.1128/mBio.00588-20 (PMC7360926; doi:10.1128/mBio.00588-20)

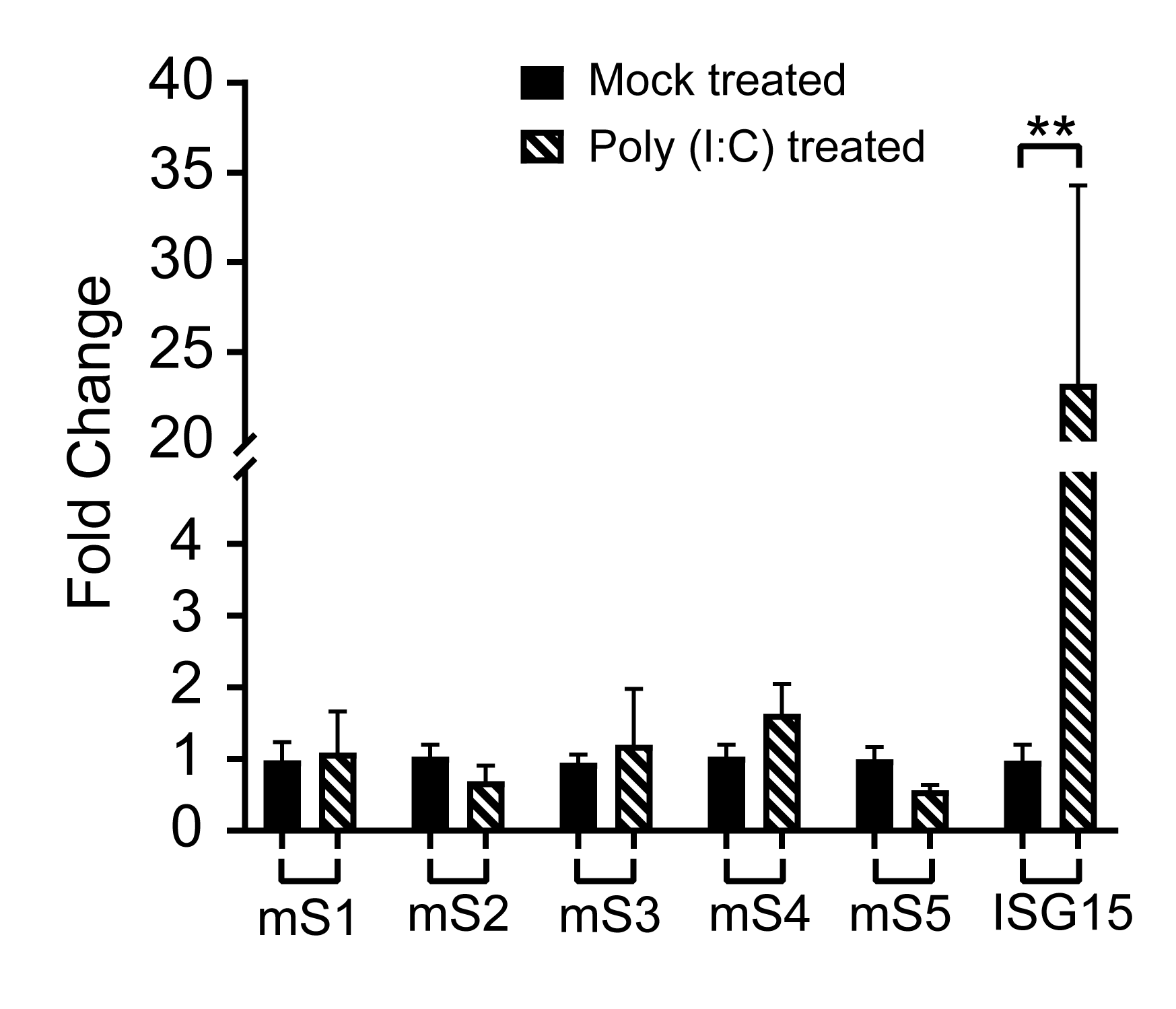

Supplement: FIG S1 [file mBio.00588-20-sf001.tif]

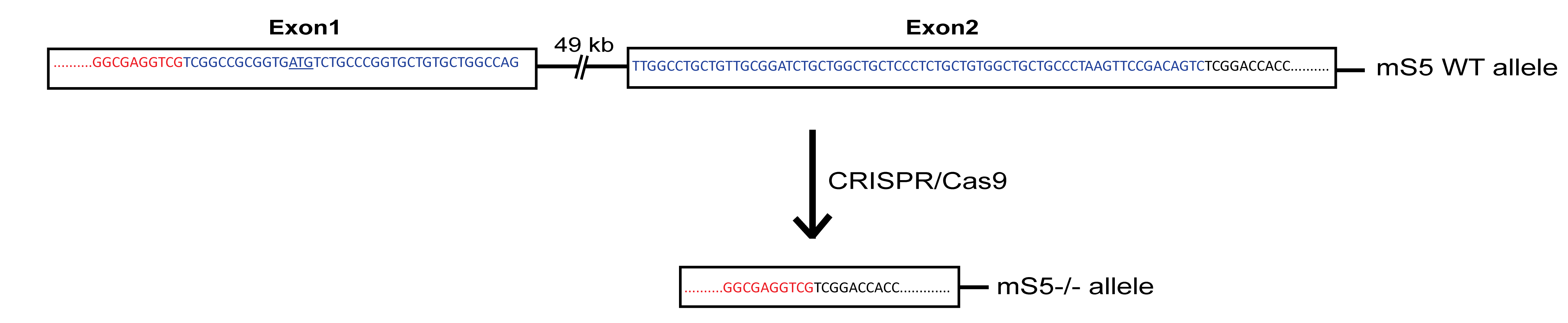

Supplement: FIG S2 [file mBio.00588-20-sf002.tif]

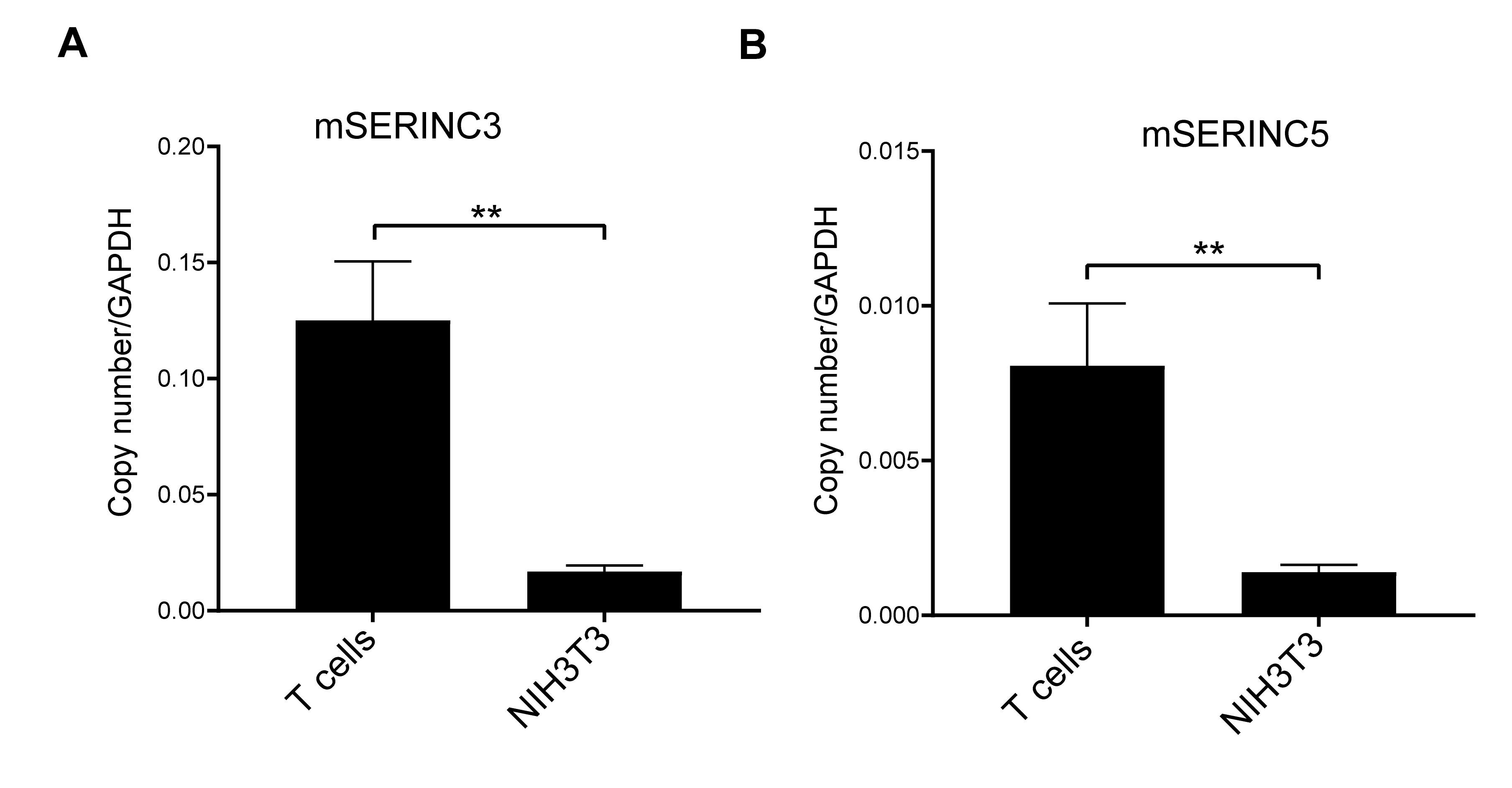

Supplement: FIG S3 [file mBio.00588-20-sf003.tif]

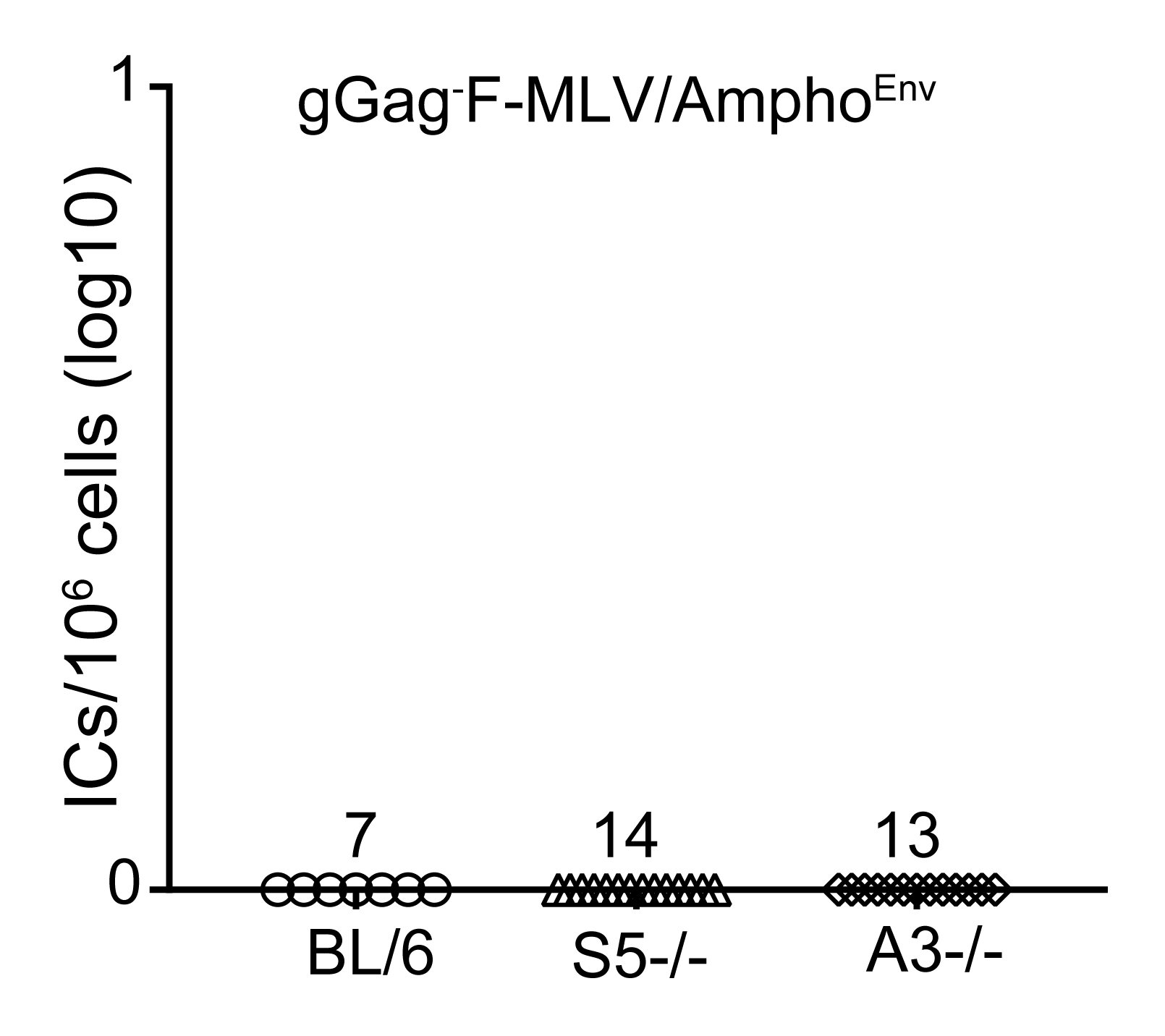

Supplement: FIG S4 [file mBio.00588-20-sf004.tif]
