# Supplementary material for: SERINC5 Potently Restricts Retrovirus Infection In Vivo
Source: mBio. 2020 Jul 14;11(4):e00588-20. doi: 10.1128/mBio.00588-20 (PMC7360926; doi:10.1128/mBio.00588-20)
Supplement: TABLE S1 [file mBio.00588-20-st001.docx]

| **S. No.** | **Name of primer** | **Nucleotide sequence** |
| --- | --- | --- |
| 1. | SEREx1F | 5’- GGGGATAGGAAGCAAGACGA-3’ |
| 2. | SEREx2R | 5’- TGCTTCATCACAGAGGGTGTC-3’ |
| 3. | SERInt2R | 5’-TCCTGGATTTATCCCACAGG-3’ |
| 4. | Serinc3_35556_F | 5’-CCCCACTGTCTGAACAAACG-3’ |
| 5. | Serinc3_35556_R | 5’-TTCCTAACACGCACATGGTTG-3’ |
| 6. | SERINC3Ex3F | 5’-CAGAGAAAGATTGTGACGTGCTG-3’ |
| 7. | SERINC3Ex3R | 5’-TGCTGCTCTGGGATCTTTACTTG-3’ |
| 8. | CAS_R1_Term | 5’-TCGTGGTATCGTTATGCGCC-3’ |
| 9. | CreF | 5’-GAACCTGATGGACATGTTCAGG-3’ |
| 10. | CreR | 5’-AGTGCGTTCGAACGCTAGAGCCTGT-3’ |
| 11. | DelF | 5’-TCCCCCTGAACCTGAAACATAA-3’ |
| 12. | DelR | 5’-TGATTTGAACTGATGGCGAGC-3’ |

**Table S1. Primers used for genotyping the mice**
