# Supplementary material for: SERINC5 Potently Restricts Retrovirus Infection In Vivo
Source: mBio. 2020 Jul 14;11(4):e00588-20. doi: 10.1128/mBio.00588-20 (PMC7360926; doi:10.1128/mBio.00588-20)
Supplement: TABLE S2 [file mBio.00588-20-st002.docx]

**Table S2. Primers used for sequencing of proviral constructs**

| **S. No.** | **Name of primer** | **Nucleotide sequence** |
| --- | --- | --- |
| 1. | FMLV gGag Sequencing_F1 | 5’-TTCGGGGGCCATTTTTGTGG-3’ |
| 2. | FMLV gGag Sequencing_R | 5’-AGCCTGGCCCATGTTTTCAG-3’ |
| 3. | FMLV gGag Sequencing_F2 | 5’-CTGGCGGATCCGTGGTGGAAC-3’ |
| 4. | FMLV Env Sequencing_F | 5’-TCTCAAAGTGGACGGCATTG-3’ |
| 5. | FMLV Env Sequencing_R | 5’-TTACTGCGGCTATCAGGCTAAGC-3’ |
